# Inositol Pyrophosphate Profiling of Two HCT116 Cell Lines Uncovers Variation in InsP_8_ Levels

**DOI:** 10.1371/journal.pone.0165286

**Published:** 2016-10-27

**Authors:** Chunfang Gu, Miranda S. C. Wilson, Henning J. Jessen, Adolfo Saiardi, Stephen B. Shears

**Affiliations:** 1 Laboratory of Signal Transduction, National Institute of Environmental Health Sciences, National Institutes of Health, 101 T.W. Alexander Drive, Research Triangle Park, North Carolina, 27709, United States of America; 2 Medical Research Council Laboratory for Molecular Cell Biology, University College London, London, United Kingdom; 3 Institute of Organic Chemistry, Albert-Ludwigs-University, Freiburg, Albertstr. 21, 79104, Freiburg, Germany; Saint George's University, UNITED KINGDOM

## Abstract

The HCT116 cell line, which has a pseudo-diploid karotype, is a popular model in the fields of cancer cell biology, intestinal immunity, and inflammation. In the current study, we describe two batches of diverged HCT116 cells, which we designate as HCT116^NIH^ and HCT116^UCL^. Using both gel electrophoresis and HPLC, we show that HCT116^UCL^ cells contain 6-fold higher levels of InsP_8_ than HCT116^NIH^ cells. This observation is significant because InsP_8_ is one of a group of molecules collectively known as ‘inositol pyrophosphates’ (PP-InsPs)—highly ‘energetic’ and conserved regulators of cellular and organismal metabolism. Variability in the cellular levels of InsP_8_ within divergent HCT116 cell lines could have impacted the phenotypic data obtained in previous studies. This difference in InsP_8_ levels is more remarkable for being specific; levels of other inositol phosphates, and notably InsP_6_ and 5-InsP_7_, are very similar in both HCT116^NIH^ and HCT116^UCL^ lines. We also developed a new HPLC procedure to record 1-InsP_7_ levels directly (for the first time in any mammalian cell line); 1-InsP_7_ comprised <2% of total InsP_7_ in HCT116^NIH^ and HCT116^UCL^ lines. The elevated levels of InsP_8_ in the HCT116^UCL^ lines were not due to an increase in expression of the PP-InsP kinases (IP6Ks and PPIP5Ks), nor to a decrease in the capacity to dephosphorylate InsP_8_. We discuss how the divergent PP-InsP profiles of the newly-designated HCT116^NIH^ and HCT116^UCL^ lines should be considered an important research opportunity: future studies using these two lines may uncover new features that regulate InsP_8_ turnover, and may also yield new directions for studying InsP_8_ function.

## Introduction

**Fig 1:**
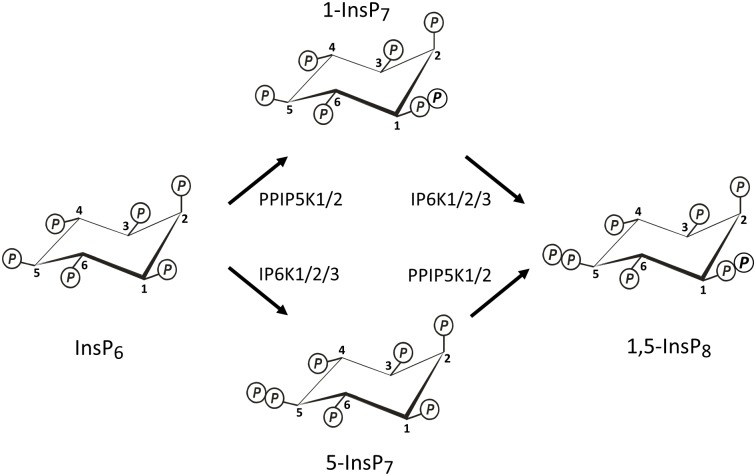
Synthesis of InsP_7_s and InsP_8_ by IP6Ks and PPIP5Ks. The Fig describes the synthesis of 1-InsP_7_, 5-InsP_7_ and 1,5-InsP_8_ in both yeasts and mammalian cells. IP6K1/2/3 = isoforms 1, 2 and 3 of inositol hexakisphosphate kinase (Kcs1 is the single yeast isoform); PPIP5K1/2 = isoforms 1 and 2 of diphosphoinositol pentakisphosphate kinase (Asp1 and Vip1 are the single isoforms in *Schizosaccharomyces pombe* and *Saccharomyces cerevisiae*, respectively).

The inositol pyrophosphates (PP-IPs; Fig 1) comprise a unique class of cell signaling molecules; crammed around a six-carbon inositol scaffold are as many as seven (“InsP_7_”) or eight (“InsP_8_”) phosphates, including functionally significant and highly ‘energetic’ diphosphate groups [1,2]. The PP-InsPs regulate many disparate biological processes, although an over-arching hypothesis has emerged that considers PP-InsPs as highly conserved regulators of cellular and organismal metabolism [1,3].

Yeasts and metazoan cells can synthesize PP-InsPs through two parallel pathways (Fig 1), which utilize two separate classes of enzymes to form diphosphate groups: the 5-kinases (the IP6Ks [4,5]) and the 1-kinases (the PPIP5Ks [6,7]). As a consequence, two InsP_7_ isomers may be generated, which are distinguished by whether the diphosphate is attached at either the 5- or 1-position on the inositol ring; InsP_8_ has both of these diphosphates (Fig 1). A family of phosphatases—the DIPPs [8]—hydrolyzes both the 1- and 5-diphosphate groups.

Research into the PP-InsPs follows a track that parallels all other investigations into the properties of intracellular signaling molecules; analyses of PP-InsP metabolism and function go hand-in-hand. Much of this work involves cultured cells, in which the levels of PP-InsPs are critical parameters that must be carefully monitored. However, such measurements can be technically challenging, due to the low (submicromolar to low micromolar) levels of PP-InsPs inside yeast and mammalian cells: steady-state concentrations of total InsP_7_ (i.e. 1-InsP_7_ plus 5-InsP_7_) lie within the 1 to 2 μM range; levels of InsP_8_ are about 10-fold lower [1,9,10]. Such measurements have traditionally been obtained by pre-labeling cells in culture with [^3^H]inositol, following which the cells are lysed, and the individual PP-[^3^H]InsPs in the soluble fraction are chromatographed by Partisphere SAX-HPLC [11,12]. To date this has been the most accurate and sensitive methodology available for monitoring cellular PP-InsP turnover. However, it does have the disadvantage of being decidedly low-throughput. For example, in order to attain steady-state labeling of PP-InsPs, mammalian cells must be incubated with [^3^H]inositol for several days [12,13]. Additionally, each HPLC run takes almost 2 h, and then the radioactivity in each individual fraction eluted from the column must be assessed by liquid scintillation counting—a total analysis time of 10 h. or more for, essentially, one experimental point [12]. Dedicated scintillation cocktail is required in order to count HPLC fractions with good efficiency at the high concentrations of salt required to elute PP-InsPs from the Partisphere SAX column. Such cocktails are expensive, as is the [^3^H]inositol itself. It is therefore not surprising that the degree of technological specialization and funding required for these experiments limits the number of laboratories that can utilize them.

Another drawback for Partisphere SAX HPLC is that it does not adequately resolve the two isomers of InsP_7_ (1-InsP_7_ and 5-InsP_7_) that are synthesized by yeast and mammalian cells [14]. In fact, as far as we are aware, there is no previous study of any mammalian cell-type in which 1-InsP_7_ has been directly quantified. Instead, the relative levels of the two isomers have only been assayed indirectly. For example, it was found that total InsP_7_ decreased about 90% upon genetic elimination of IP6K2 [15], or by inhibition of IP6K activity by a cell-permeant pan-IP6K inhibitor, *N*2-(*m*-(trifluoromethyl)benzyl) *N*6-(P-nitrobenzyl)purine [16]. Neither study confirmed that the synthesis of 5-InsP_7_ was completely eliminated, but at least it was possible to conclude that 1-InsP_7_ comprises no more than 10% of total InsP_7_. However, there remains a need to assay cellular 1-InsP_7_ levels directly, particularly in view of its distinct role as a pro-inflammatory mediator [17].

Recently, a gel electrophoresis method was developed for assaying cellular PP-InsPs; this procedure does not rely on [^3^H]inositol labeling, is far less costly, and has much higher throughput [18–20]. All of the required equipment should be routinely available in any biochemical research laboratory. Consequently, an increasing number of laboratories now have the capability to study PP-InsP metabolism. This method does not match the sensitivity of HPLC, but by using TiO_2_ beads to concentrate PP-InsPs prior to analysis [19], the cellular levels of total InsP_7_ can be readily monitored. This methodology can even resolve 5-InsP_7_ from 1-InsP_7_ [20], but to date gel electrophoresis has not detected 1-InsP_7_ in any mammalian cell line [19], perhaps because its levels are below the limits of sensitivity. Thus, we have developed an alternative HPLC technique that, for the first time, can directly measure 1-InsP_7_ levels in intact cells.

The assay of cellular InsP_8_ has also proved to be challenging for gel electrophoresis, at least when using an experimentally convenient number of cells [19]. However, a recent analysis of an HCT116 human colonic carcinoma cell line revealed it to contain substantially higher levels of InsP_8_ than those found in some other mammalian cell lines [19]. We now demonstrate that there is considerable variability in the cellular levels of InsP_8_ within two divergent HCT116 cell lines in our two laboratories. We designate the two lines as HCT116^NIH^ (containing ‘low’ InsP_8_ levels) and HCT116^UCL^ (containing ‘high’ InsP_8_ levels). We discuss the wider significance of this difference in the levels of a key component of the multi-functional PP-InsP signaling cascade in the two HCT116 cell line variants.

## Materials and Methods

### Cell culture and [^3^H]inositol radiolabeling

The HCT116 lines that have been used by the NIH and UCL laboratories are designated as HCT116^NIH^ and HCT116^UCL^, respectively; both originate from ATCC. The HCT116^NIH^ cells were provided as frozen stocks that were obtained in 1994 by the laboratory of Dr Thomas Kunkel at NIEHS [21]. The HCT116^UCL^ cells were provided as frozen stocks that were obtained in 2012 by the laboratory of Dr Sibylle Mittnacht at UCL. In addition, a batch of HCT116 cells was procured directly from ATCC. Cells were cultured for less than 15 passages, and the data obtained were independent of this time in culture. For experiments that were performed in the NIH laboratory, all cells were cultured under identical conditions in DMEM/F12 medium (ThermoFisher Scientific) supplemented with 10% Fetal Bovine serum (Germini Bio-product) and 100 U/ml Penicillin-Streptomycin (ThermoFisher Scientific) at 37°C, 5% CO_2_. For experiments that were performed in the UCL laboratory, all cells were cultured under identical conditions in DMEM medium (ThermoFisher Scientific) supplemented with 10% Fetal Bovine serum (Sigma) at 37°C, 5% CO_2_. All cultures in both the UCL and NIH laboratories were tested for mycoplasma using the MycoAlert^™^ kit; no mycoplasma was detected.

To measure cell growth, 2x10^5^ cells were seeded in 6-well plates with 2 ml culture medium and cultured for 4 days. Each day, cells in one plate were trypsinized and counted using a Countess I (ThermoFisher Scientific).

For the radiolabeling experiments, 1x10^6^ cells were seeded in a 10 cm dish with 7 ml medium supplemented with 10 μCi/ml [^3^H]inositol. After 3 days of radiolabeling, at which point cultures were 60% to 70% confluent, the cells were quenched by removal of the culture medium and its immediate replacement with 1 ml of ice-cold 1M perchloric acid (the yield of PP-InsPs is very similar when using alternative, non-acidic quench techniques, i.e. at pH 7.7 [11]). The plates were placed on ice for 15 min, then the soluble portion was taken for HPLC analysis of the PP-[^3^H]InsPs (see below). The insoluble cell debris was solubilized in 8 ml of 0.1 M NaOH / 0.1% triton X-100 overnight, after which aliquots were taken to assess total [^3^H]inositol lipids, for normalizing the levels of each of the [^3^H]inositol phosphates.

### HPLC analysis of cellular inositol phosphates

Inositol phosphates were resolved by HPLC using either a 4.6 x 125 mm Partisphere SAX HPLC column (Whatman), or a 3 x 250 mm CarboPac^™^ PA200 HPLC column (ThermoFisher Scientific).

Acid-quenched cell extracts that were to be chromatographed on a Partisphere SAX column were neutralized with 675 μl of ice-cold 1M KCO_3_ / 40 mM EDTA. After 15 min on ice, the perchlorate pellet was removed by centrifugation, and the supernatant was diluted 1:1 with 1 mM Na_2_EDTA. Samples were loaded onto the HPLC column and eluted with a gradient that was generated from Buffer A (1 mM Na_2_EDTA) and Buffer B (Buffer A plus 1.3 M (NH_4_)_2_HPO_4_, pH 3.85 with phosphoric acid). The gradient (1 ml/min) is as follows: 0–10 min, 0% B; 10–25 min, B increased linearly from 0 to 35%; 25–105 min, B increased linearly from 35 to 100%. From each run, 1 ml fractions were collected and vigorously mixed with 4 ml MonoFlow 4 (National Diagnostics, Manville NJ), and the [^3^H] DPM/fraction was measured with a liquid scintillation counter.

For acid-quenched cell extracts that were to be chromatographed on a CarboPac^™^ PA200 HPLC column, 1.5 mg titanium dioxide (TiO_2_) beads (Titansphere TiO 5 mm; GL Sciences) were added so as to bind the inositol phosphates [19]; samples were rotated at 4°C for 30 min. The beads were concentrated by centrifugation, and washed twice with ice-cold water. Inositol phosphates were eluted from the beads by sequential washes in 1 ml and then 0.5 ml of 1.5 M ice-cold NH_4_OH; each time, samples were rotated at 4°C for 20 min. The two supernatants were combined, and vacuum evaporated to approx. 50 μl. Next, each sample was spiked with 1 nmol of InsP_6_ (Calbiochem), and 1 nmol each of chemically synthesized 1-InsP_7_ [22], 5-InsP_7_ [23], and InsP_8_ [24]. For some experiments (as indicated below), 20 nmol 5-InsP_7_ were added. Samples were made up to 230 μl with Buffer C (1 mM Na_2_EDTA, 10 mM 1,4-piperazinedipropanesulfonic acid, pH 4.7, 5% MeOH), loaded on to the HPLC column and eluted with a gradient that was generated from Buffer C and Buffer D (Buffer C plus 0.5 M tetramethylammonium nitrate (Sigma-Aldrich)). The gradient (0.5ml/min) is as follow: 0- 10min, 0% D; 10-15min, D increased linearly from 0% to 30%; 15-60min, D increased linearly from 30% to 55%; 60-70min, D increased linearly from 55% to 65%. From each run 0.25ml fractions were collected, mixed with 3 ml MonoFlow 4, and the [^3^H] DPM/fraction was measured with a liquid scintillation counter.

### InsP_8_ phosphatase activity assay

60%-70% confluent cells in 10 cm dishes were scraped into 10 ml ice-cold PBS. Cell pellets were lysed for 15 min on ice in 150 μl buffer (20 mM Tris pH7.5, 150 mM NaCl, 5% glycerol, 0.5% Triton X-100) and then homogenized using a Minilys personal homogenizer (Precellys). Protein concentration was measured using a BCA protein assay kit (ThermoFisher Scientific). Next, 70 μg cell lysate (10 μl) was incubated with 1 μM [^3^H]InsP_8_ in 100 μl assay buffer (1 mM Na_2_EDTA, 20 mM 4-(2-hydroxyethyl)-1-piperazineethanesulfonic acid, 100 mM KCl, 2 mM MgCl_2_) at 37°C for 0 or 10min. Reactions were quenched with perchloric acid followed by neutralization with K_2_CO_3_. After centrifugation, the supernatants were applied to a Partisphere SAX HPLC using a gradient generated from Buffers A and B as described above, with slight modificaton: 0–5 min, 0% D; 5–10 min, D increased linearly from 0 to 45%; 10–60 min, D increased linearly from 45% to 100%. From each run 1 ml fractions were collected and mixed with 4 ml MonoFlow 4 scintillant.

### Gel electrophoresis and visualization of inositol phosphates

For the PAGE experiments, 8 x 10^6^ cells were seeded into 6 x 15 cm dishes with 18 ml medium. After 3 days of growth, at which point cultures were 90% confluent, the cells were trypsinised and washed in PBS. Extracts were made from 6 x 10^7^ cells (DAPI staining) or 8 x 10^7^ cells (toluidine staining). Cells were extracted in 1 ml 1 M perchloric acid, as described previously [19], using 5 mg TiO_2_ beads per sample. Inositol phosphates were eluted using 2.5% NH_4_OH, and separated using 35% PAGE and visualized with DAPI or toluidine blue as described [20].

### Western blot analysis

Cells that were to be analyzed by Western blotting were seeded (0.4 x 10^6^ cells) into 6-well plates and harvested 48 later, at which point they were 80% confluent. Cells were lysed in RIPA buffer containing Halt^™^ Protease and Phosphatase Cocktail (ThermoFisher Scientific) and further homogenized using a Minilys personal homogenizer. Typically, 20 μg of protein was loaded onto an SDS-PAGE gel for immunoblotting. Primary polyclonal antibodies used: IP6K1 (Prestige HPA040825, 1:1000, Sigma), IP6K2 (sc-10425, 1:1000, Santa Cruz Biotech), PPIP5K2 (ab154046, 1:1000, Abcam), b-actin (sc-1615 HRP, 1:10000, Santa Cruz Biotech). Detection was performed using Luminata Crescendo Western HRP Substrate (Millipore) or SuperSignal West Femto Kit (Thermo Scientific) for IP6K2. The antibodies against IP6K1 and IP6K2 were validated (see S1 Fig) with the help of mouse embryonic fibroblasts derived from IP6K1^-/-^ mice [25], and IP6K2^-/-^ HCT116 cells [15]; both of the latter cell-lines were kindly provided by Solomon Snyder. The antibody against PPIP5K2 also cross-reacts with PPIP5K1. To validate this antibody (see S2 Fig), we created HCT116^NIH^ cell lines in which expression of either PPIP5K1 or PPIP5K2 was eliminated by using CRISPR [26].

### Microscopy

For morphology analysis, 8 x 10^4^ cells were seeded onto glass coverslips in a 12 well plate. After 3 days of growth, at which point cultures were 50% confluent, the cells were fixed in 4% formaldehyde for 10 min. Cells were then permeabilised in 0.2% Triton-X100 for 10 min, blocked with 10% goat serum for 1 h, then stained with 0.4 μM FITC-phalloidin (Sigma) and 10 μg/ml Hoechst 33342 for 1 h. Confocal microscopy was performed using a Leica SPE microscope with a 63x lens. Images shown are maximal projections of Z-stacks.

### Quantitative Reverse Transcription PCR

Cells that were to be analyzed by RTq-PCR were seeded (0.4 x 10^6^ cells) into 6-well plates and harvested 48 later, at which point they were 80% confluent. RNA was extracted from cells using RNeasy Kit (QIAGEN), and converted to cDNA with SuperScript III First Strand Synthesis System (Invitrogen). The qRT-PCR was performed using MESA Blue qPCR mix (Eurogentec) in a Mastercycler ep Gradient S (Eppendorf). Results were normalized to a standard curve of purified IP6K CDS of known copy number. The following primer pairs were used: IP6K1, forward GAGGAGAAAGCCAGCCTGT, reverse TTCTCAAGCAGGAGGAACTTG; IP6K2 forward AGTCATTGGTGTGCGTGTGT, reverse ACCAGCAGGGAGCTTGAGTA; IP6K3 forward AAGACACCAACGGAAACCAG, reverse, AGATCCAGGACACAGGGATG.

## Results and Discussion

### Analysis of PP-InsP profiles in HCT116 cells using gel electrophoresis and Partisphere SAX HPLC

In a recent study from the UCL laboratory [19], gel electrophoresis was used to determine the levels of InsP_6_, InsP_7_, and InsP_8_ in HCT116 cells (re-designated here as HCT116^UCL^ cells). The levels of InsP_8_ in these cells (computed as a ratio to InsP_6_) were shown to be approx. 10-fold higher than those in several other mammalian cell types: HeLa, CHO, HT29, PC3, and MCF7 [19].

**Fig 2:**
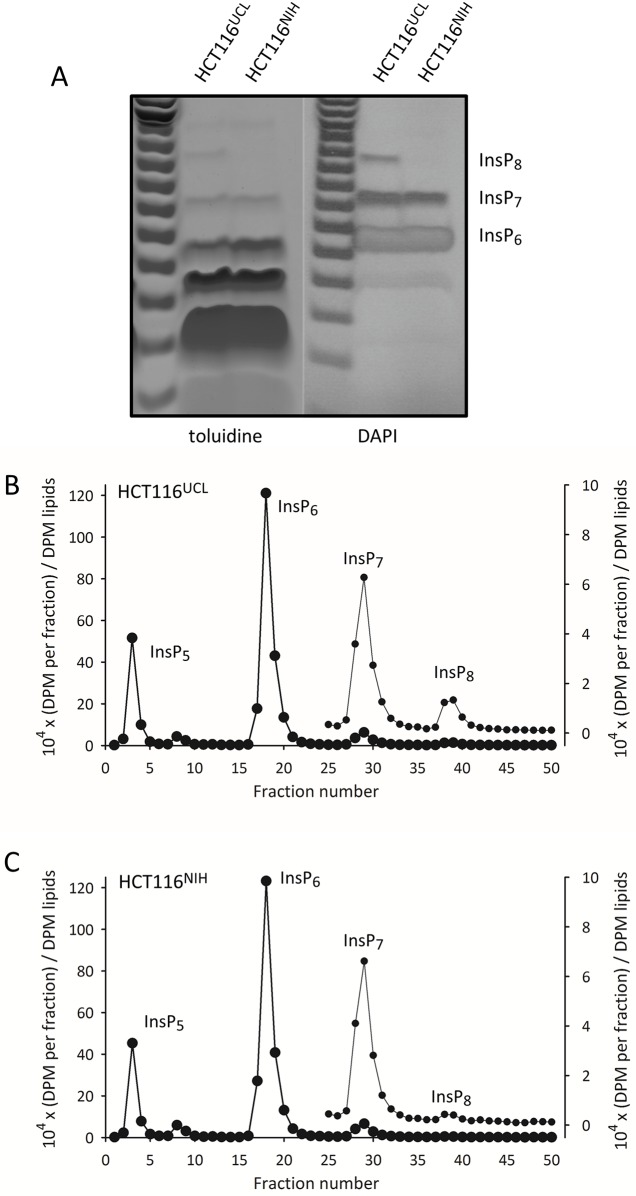
Differences in InsP_8_ levels between HCT116^UCL^ and HCT116^NIH^ cells. Panel A: extracts of HCT116^NIH^ and HCT116^UCL^ cells were prepared by using TiO_2_ to concentrate inositol phosphates, which were then resolved by electrophoresis on a 35% polyacrylamide gel, and visualized by staining with either toluidine or DAPI. Panels B,C show Partisphere SAX HPLC analysis of extracts of [^3^H]inositol-labeled HCT116^UCL^ cells and HCT116^NIH^ cells, respectively. The DPM in each fraction were normalized to the DPM (x10^4^) of the [^3^H]inositol lipids. Fractions 25–50 are re-plotted on an expanded scale (left-hand axis), to highlight the InsP_7_ and InsP_8_ peaks.

We have now compared the levels of InsP_6_, InsP_7_, and InsP_8_ in HCT116^UCL^ cells with those in a different batch of HCT116 cells in use in the NIH laboratory (now re-designated as HCT116^NIH^ cells). Both sets of cells were cultured and analyzed in the UCL laboratory under identical conditions. Levels of InsP_6_ and total InsP_7_ are very similar in both groups of cells, but the levels of InsP_8_ are substantially higher in the HCT116^UCL^ cells (Fig 2A). The difference in InsP_8_ levels were not quantified precisely by gel electrophoresis, as the signal from the HCT116^NIH^ cells is below the level of detection (Fig 2A).

We next performed an alternative, and more sensitive assay of cellular PP-InsPs, using Partisphere SAX-HPLC analysis of extracts prepared from [^3^H]inositol labeled cells. For these experiments, HCT116^UCL^ and HCT116^NIH^ cells were cultured and analyzed in the NIH laboratory under identical conditions. The level of [^3^H]InsP_8_ in HCT116^UCL^ cells (Fig 2B) was found to be about 6-fold higher than its level in HCT116^NIH^ cells (Fig 2C). Again, levels of InsP_6_ and total InsP_7_ were similar in both cell types. Thus, we conclude that these two populations of cells have diverged in a very specific aspect of PP-InsP turnover: the regulation of InsP_8_ levels.

### Validation of the lineage of the HCT116^NIH^ and HCT116^UCL^ lines

We considered it important to validate that neither batch of HCT116 cells in our two laboratories might be misidentified, such as does occur with surprising frequency, as a consequence of mislabeling or by contamination with another cell line [27]. Cell line authenticity was interrogated by PCR amplification of amelogenin plus tandem DNA repeat sequences (STRs) at eight core alleles, using the ATCC profiling service. These data were compared with those for the HCT116 cell line (catalogue number CCL-247) that is curated by ATCC (Table 1). Both the HCT116^NIH^ and HCT116^UCL^ lines exceed the 80% allele match that is considered sufficient to designate common ancestry [27]. The power of discrimination for this analysis has been estimated to be approximately 1 x 10^-8^ [27]. Thus, we conclude that neither of our two cell lines have been misidentified or contaminated by other lines.

**Table 1:** STR profiles of HCT116^NIH^ and HCT116^UCL^ cell-lines, compared with HCT116 cells curated at ATCC. The loci for eight core short tandem repeats plus Amelogenin were derived by ATCC for their curated HCT116 cell line (catalogue number CCL-247) and the HCT116^NIH^ and HCT116^UCL^ cells. The HCT116^NIH^ and HCT116^UCL^ cells had an 83% and 89% match with the parental HCT116 line, above the 80% minimum that designates common lineage.

		Repeat number	
Alleles	HCT116 (ATCC)	HCT116^NIH^	HCT116^UCL^
D5S818	10,11	10,11	10,11,12
D13S317	10,12	10,12	10,12
D7S820	11,12	10,12	11,12
D16S539	11,13	11,13	11,12,13
vWA	17,22	18,19,21	17,22
THO1	8,9	8,9	8,9
AMEL	X,Y	X,Y	X
TPOX	8,9	8,9	8
CSF1PO	7,10	7,10	7,10

**Fig 3:**
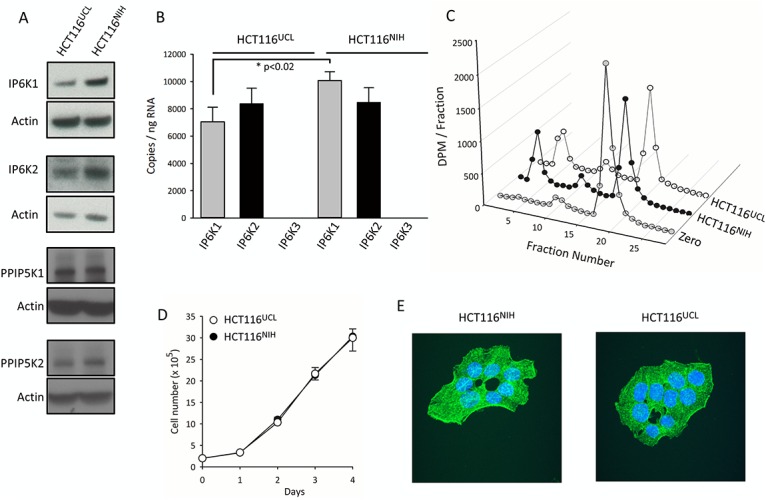
Comparisons of HCT116^NIH^ and HCT116^UCL^ cells: expression of IP6Ks and PPIP5Ks, capacity to dephosphorylate InsP_8_, cell growth, and phalloidin staining. The following analyses of HCT116^NIH^ and HCT116^UCL^ cells were performed: Panel A, Western analyses of IP6Ks and PPIP5Ks. Complete gels, and procedures used to validate the antibodies, are described in S1 and S2 Figs. Panel B, quantitative RT-PCR analysis of expression of *IP6K1*, *IP6K2* and *IP6K3*. Panel C, HPLC analysis of 1 μM [^3^H]InsP_8_ dephosphorylation by 70 μg cell lysates in 100 μl medium. Panel D, counting of cell growth for the indicated number of days. Panel E, labeling of the actin cytoskeleton with FITC-phalloidin. Hoechst was used as a nuclear stain.

Nevertheless, as neither allele match was 100%, both of the cell lines were deemed to have undergone some genomic changes. Indeed, it is known (yet frequently ignored [28]) that all tumor-derived cell lines suffer from varying degrees of inherent genomic instability which can promote divergence [29,30]. We propose that genomic changes underlie the stable differences in InsP_8_ levels between these two HCT116 cell-lines. We therefore investigated if the HCT116^UCL^ cell-lines might express higher levels of the kinases—IP6Ks and PPIP5Ks—that synthesize the PP-InsPs. This was not the case according to Western analysis of the expression of IP6K1, IP6K2, PPIP5K1, and PPIP5K2 (Fig 3A). In fact, there is an indication that the HCT116^UCL^ cells express slightly *lower* levels of IP6K1 than do the HCT116^NIH^ cells (Fig 3A); the latter result is opposite to that which might have helped account for the higher levels of InsP_8_ in the HCT116^UCL^ line. Specific antibodies against IP6K3 were not available, so we examined expression of the IP6Ks by qRT-PCR. Neither cell line expressed IP6K3 (Fig 3B). This analysis also confirmed a slightly lower level of expression of IP6K1 in HCT116^UCL^ cells.

We also conducted experiments to investigate if the two HCT116 cell line variants might differ in their rates of InsP_8_ dephosphorylation. This is a complex topic, for several reasons. First, there is a group of InsP_8_ phosphatases in mammals—the DIPPs—that comprise 5 different isoforms that each have slightly differing kinetic parameters [14]. We do not have antibodies that can distinguish between all of these different DIPPs. Two of these enzymes—DIPP2α and DIPP2β—are generated from an array of alternately spliced mRNAs that may have differential stability and translatability. InsP_8_ is also dephosphorylated by a phosphatase domain in the PPIP5Ks [31]. Finally, the discovery of a new PP-InsP phosphatase in yeast [32] raises the possibility that additional mammalian InsP_8_ phosphatases remain to be discovered. In such circumstances, we measured total InsP_8_ dephosphorylation in cell lysates prepared from HCT116^NIH^ cells and HCT116^UCL^ cells (Fig 3C), and found no substantial difference between them.

We further found that the HCT116^NIH^ and HCT116^UCL^ cell-lines exhibited identical growth-rates (Fig 3D), and they exhibit similar morphological organization that could not be distinguished by phalloidin staining of the actin cytoskeleton (Fig 3E).

### Analysis of PP-InsP profiles in HCT116 cells using a CarboPac HPLC system

There are two parallel pathways to InsP_8_ synthesis, each of which use different InsP_7_ isomers as intermediates: 5-InsP_7_ and 1-InsP_7_ (Fig 1). We posited that information on the relative levels of the two InsP_7_ precursors may inform on the manner in which InsP_8_ accumulation is up-regulated in HCT116^UCL^ cells as compared to HCT116^NIH^ cells. However, it has not previously been possible to directly compare cellular 5-InsP_7_ and 1-InsP_7_ levels: gel electrophoresis is not sufficiently sensitive, and Partisphere SAX HPLC does not have the resolution capability [14]. There is an alternative, mass-detection HPLC method that can separate the two InsP_7_s, but again it lacks the required sensitivity [6]. In any case, the latter method utilizes an HCl-based mobile phase that, at ambient temperature, may cause PP-InsP degradation [18,33]. To date, 1-InsP_7_ levels have only been estimated indirectly, either by using genetic manipulations [15] or pharmacological tools [16] to reduce, but not definitively eliminate, the synthesis of 5-InsP_7_.

**Fig 4:**
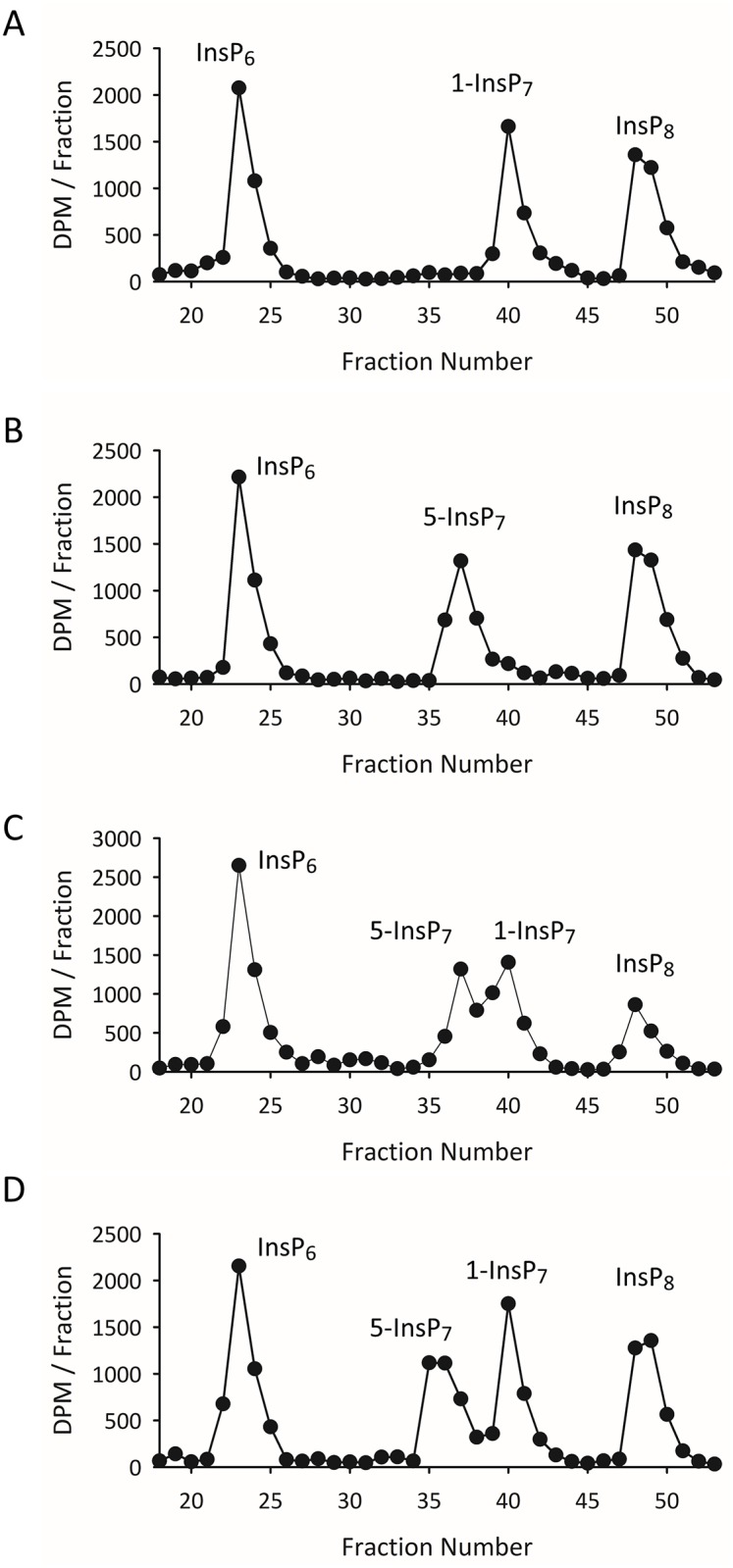
Separation of 1-InsP_7_ and 5-InsP_7_ by CarboPac HPLC. Standards of [^3^H]InsP_6_, 1-[^3^H]InsP_7_, 5-[^3^H]InsP_7_, and [^3^H]InsP_8_ (1 nmol of each) were chromatographed on a CarboPac HPLC column. Panels A and B show HPLC runs in which either 1-[^3^H]InsP_7_ or 5-[^3^H]InsP_7_ were added individually, while Panel C shows an HPLC run in which both [^3^H]InsP_7_ isomers were added together. Panel D, the mass amount of 5-InsP_7_ was increased to 20 nmol.

In the current study we have resolved the 1-InsP_7_ from 5-InsP_7_ using an alternative HPLC protocol (Fig 4) that uses a CarboPac column [31]. Unlike the Partisphere SAX column, the CarboPac HPLC column has mixed-mode separation characteristics: quaternary amines for the anion-exchange phase are attached in a low capacity format to poly(styrene-divinylbenzene) for reverse-phase chromatography [34]. The ability of these columns to resolve inositol phosphate isomers was first reported in 2003 [35], but that study used an HCl gradient at room temperature, which likely would degrade PP-InsPs [19,33]. Instead, we eluted at pH 4.7. To minimize cation interactions with PP-InsPs while maximizing anion-interactions, we eluted with tetramethylammonium nitrate [36] in the presence of 5% methanol as an organic modifier. Peak sharpness was enhanced by adding non-radioactive ‘spikes’ of 1-InsP_7_ and 5-InsP_7_. The use of individual radioactive standards shows clear separation of InsP_6_, 1-InsP_7_, 5-InsP_7_ and InsP_8_ (Fig 4A and 4B). Recoveries of each standard exceeded 85%; the small losses were largely intrinsic to the handling of the materials.

**Fig 5:**
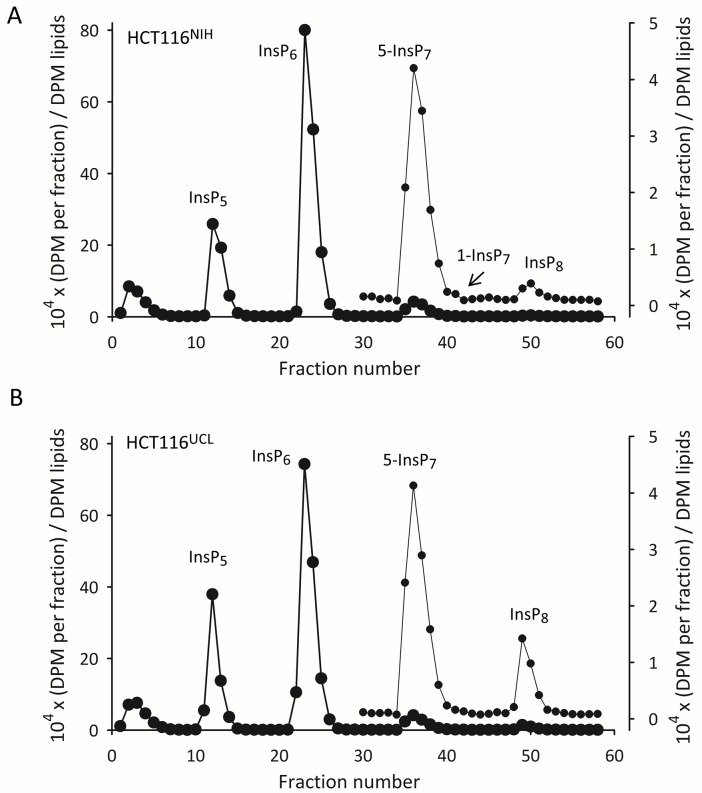
CarboPac HPLC analysis of [^3^H]inositol-labeled inositol phosphates in HCT116^NIH^ and HCT116^UCL^ cells. Extracts of [^3^H]inositol-labeled HCT116^NIH^ cells (Panel A) and HCT116^UCL^ cells (Panel B) were prepared in parallel and analyzed by CarboPac HPLC. The DPM in each fraction were normalized to the DPM of the [^3^H]inositol lipids. Fractions 30–58 are re-plotted on an expanded scale (left-hand axis), so as to highlight the InsP_7_ and InsP_8_ peaks. This experiment was performed six times. In the experiment shown, 1-InsP_7_ is only discernable in the HCT116^NIH^ cells. S3 Fig shows a separate experimental pair in which 1-InsP_7_ was only observed in the HCT116^UCL^ cells.

When standards of 1-InsP_7_ and 5-InsP_7_ were chromatographed together, their partial separation was confirmed (Fig 4C). Furthermore, when the mass of the 5-InsP_7_ spike was increased, the resolution of the two InsP_7_ isomers was significantly improved (Fig 4D). We used this HPLC protocol to resolve extracts prepared from [^3^H]inositol-labeled HCT116^UCL^ and HCT116^NIH^ cells that were radiolabeled in parallel. We were surprised to discover that, for each cell-line, a distinct 1-InsP_7_ peak was observed in just one of six HPLC runs. In the experiment described by Fig 5A and 5B, 1-InsP_7_ is only discernable in the HCT116^NIH^ cells. S3 Fig shows a separate experimental pair in which 1-InsP_7_ was only observed in the HCT116^UCL^ cells. In each case that 1-InsP_7_ was clearly distinguished, it amounted to just 1.5 to 2% of total InsP_7_.

The rest of the data obtained from the Carbopac column are consistent with those obtained from the Partisphere SAX column (Fig 2B and 2C) in that the levels of InsP_5_, InsP_6_ and 5-InsP_7_ are very similar in the two cell lines, while the HCT116^UCL^ cells contain approx. 6-fold higher levels of InsP_8_ (Fig 5; S4 Fig). We also performed HPLC analysis of a new batch of HCT116 cells that we acquired direct from ATCC. The levels of InsP_8_ in these cells, recorded after 2 and 10 passages, were very similar to those of HCT116^NIH^ cells (S4 Fig).

## Concluding Comments

The possibility of diverse phenotypes in a cell line used by multiple laboratories is a subject that receives little attention in the scientific literature. The current study is therefore unusual in that it describes two HCT116 cell line variants, designated HCT116^NIH^ and HCT116^UCL^, that are phenotypically distinguishable by virtue of their significantly different levels of InsP_8_ (Figs 2 and 5). The observation was confirmed using Partisphere SAX HPLC, Carbopac HPLC, and gel electrophoresis. This difference in InsP_8_ levels between two cell lines of common ancestry is all the more remarkable for being specific; levels of other inositol phosphates, and notably InsP_6_, 1-InsP_7_ and 5-InsP_7_, are very similar in both cell lines. This divergence has occurred despite HCT116 cells being among the more genomically stable of colorectal lines [29,30]. Nevertheless, our study underscores how any cell line is potentially susceptible to genetic divergence, as a consequence of subtle differences in culture conditions such as the nature of the medium, serum concentration, temperature, humidity, and other cell-handling practices.

During the time that has passed since the isolation of a homogeneous culture of HCT116 cells from a single human colonic carcinoma, 35 years ago [37], 9316 articles can be retrieved from the PubMed archive by using “HCT116 or HCT-116" as a query (as of August 10, 2016). Moreover, HCT116 cells have been utilized by many cancer cell biologists [38], and are also employed as a model for studying intestinal immunity and inflammation [39]. These very biological phenomena are among those known to be regulated by members of the PP-InsP family [17,40]. That is, the cell line is a particularly appropriate model for PP-InsP research. A key mechanism by which PP-InsPs regulate cell function is by a non-enzymatic, concentration dependent pyrophosphorylation of a wide range of proteins [41,42]. The 6-fold disparity in InsP_8_ levels between HCT116^NIH^ and HCT116^UCL^ cells represents a significant variation in the pyrophosphorylation capacity of the two different lines. It is very possible that differences in the cellular levels of InsP_8_ could alter the phosphorylation profile of multiple proteins, impacting the biological data obtained with the HCT116 cells used in earlier studies. Future work with HCT116 cells should consider taking this variability into account by profiling PP-InsP levels.

Nevertheless, the elevated levels of InsP_8_ in HCT116^UCL^ cells also represent a research opportunity. Among the members of the PP-InsP signaling family, InsP_8_ is the one that shows the most acute changes in cellular levels in response to certain extracellular and intracellular perturbations. For example, InsP_8_ levels increase several-fold when cells are subjected to defined environmental challenges, such as hyperosmotic stress [43], heat stress [44], and cold stress [44]. InsP_8_ also appears to act as a metabolic sensor, since its levels decrease in cells undergoing relatively mild bioenergetic challenges, even those that can occur in the absence of a detectable drop in ATP levels [45]. For future studies that investigate PP-InsP metabolism and function, it may be useful that the elevated levels of InsP_8_ in HCT116^UCL^ cells bring them into the range of values that can be readily monitored by gel electrophoresis, which is more experimentally friendly than is HPLC.

Our study also provides the first direct determination of the cellular level of 1-InsP_7_ in any mammalian cell-line. It is of further significance that 1-InsP_7_ accounts for less than 2% of total InsP_7_, a level that was only detected in one of six HPLC runs (Fig 4, S3 Fig). Thus, the 6-fold higher accumulation of 1,5-InsP_8_ in HCT116^UCL^ cells is not accompanied by a significantly increased 1-InsP_7_ synthesis. That is, it seems unlikely that the 1-kinase *activities* of PPIP5Ks (Fig 1) is substantially higher in the HCT116^UCL^ cells compared to the HCT116^NIH^ cells, consistent with there being similar *levels* of these enzymes in the two groups of cells (Fig 3A). It remains to be determined how the extremely low levels of 1-InsP_7_ impact ideas concerning its proposed signaling activities. For example, it has been reported to have pro-inflammatory properties [17]; perhaps 1-InsP_7_ levels increase in response to certain pathogenic challenges. A wider application of the CarboPac HPLC method would appear to be essential for any future research that might specifically study the metabolism and function of 1-InsP_7_. Finally, by demonstrating that the levels of InsP_8_ are substantially different in two variants of a particular cell line, our data indicate the importance for future work in the PP-InsP field of validating cellular PP-InsP content by either HPLC or gel electrophoresis—whichever cell type is used.

## Supporting Information

S1 Fig Full Western blots for IP6Ks, and antibody validation. Panel A, complete blots are shown for the Western analyses of levels of IP6K1, IP6K2 and actin as depicted in Fig 3A of the main text. Panel B, validation of the band detected by the anti-IP6K2 antibody (using an extract prepared from IP6K2^-/-^ HCT116 cells) and the anti-IP6K1 antibody (using an extract prepared from IP6K1^-/-^ MEF cells).
(PPTX)

S2 Fig Full Western blots for PPIP5Ks, and antibody validation. Panels A, B, complete blots are shown for the Western analyses of levels of PPIP5K2, PPIP5K1 and actin as depicted in Fig 3A of the main text. Panel C, validation of the PPIP5K1 and PPIP5K2 band detected by the anti-PPIP5K2 antibody, in a single blot with two different exposure times. K1KO and K2KO lanes show extracts prepared from cells in which either PPIP5K1 or PPIP5K2 expression, respectively, was eliminated using CRISPR. Single-guide RNAs(sgRNA) with sequences 5’-CCCCTTTCTTATCAATGATCTGG-3’ and 5’-CGGTTCAAAATAGCATAACGAGG-3’ were designed to target PPIP5K1 exon 4 and PPIP5K2 exon 5 respectively. Vector expressing both cas9 and sgRNA was obtained from Addgene (PX458). PPIP5Ks KO cells were generated following the protocol as described: *Genome engineering using the CRISPR-Cas9 system*. Nat Protoc. 2013 Nov; 8(11): 2281-308. doi: 10.1038/nprot.2013.143. Epub 2013 Oct 24.
(PPTX)

S3 Fig Analysis by CarboPac HPLC of [^3^H]InsP_7_ and [^3^H]InsP_8_ in HCT116^NIH^ and HCT116^UCL^ cells. Extracts of [^3^H]inositol-labeled HCT116^NIH^ cells (Panel A) and HCT116^UCL^ cells (Panel B) were prepared in parallel and analyzed by CarboPac HPLC. The DPM in each fraction were normalized to the DPM of the [^3^H]inositol lipids. Only InsP_7_ and InsP_8_ peaks are shown. This experiment was performed six times. In the experiment shown, 1-InsP_7_ is only discernable in the HCT116^UCL^ cells. Fig 5 in the main text shows a separate experimental pair in which 1-InsP_7_ was only observed in the HCT116^NIH^ cells.
(PPTX)

S4 Fig [^3^H]InsP_8_ levels in individual HCT116 lines. CarboPac HPLC was used to quantify [^3^H]InsP_8_ levels in extracts prepared from [^3^H]inositol-labeled HCT116^NIH^ cells, HCT116^UCL^ cells, and also parental HCT116 cells that were procured directly from ATCC and analyzed after 2 passages (“2p”) and 10 passages (“10p”). [^3^H]InsP_8_ levels are normalized to those of [^3^H]InsP_6_.
(PPTX)
