# Supplementary figures and images for: Inositol Pyrophosphate Profiling of Two HCT116 Cell Lines Uncovers Variation in InsP8 Levels
Source: PLoS One. 2016 Oct 27;11(10):e0165286. doi: 10.1371/journal.pone.0165286 (PMC5082907; doi:10.1371/journal.pone.0165286)

## Slide 1
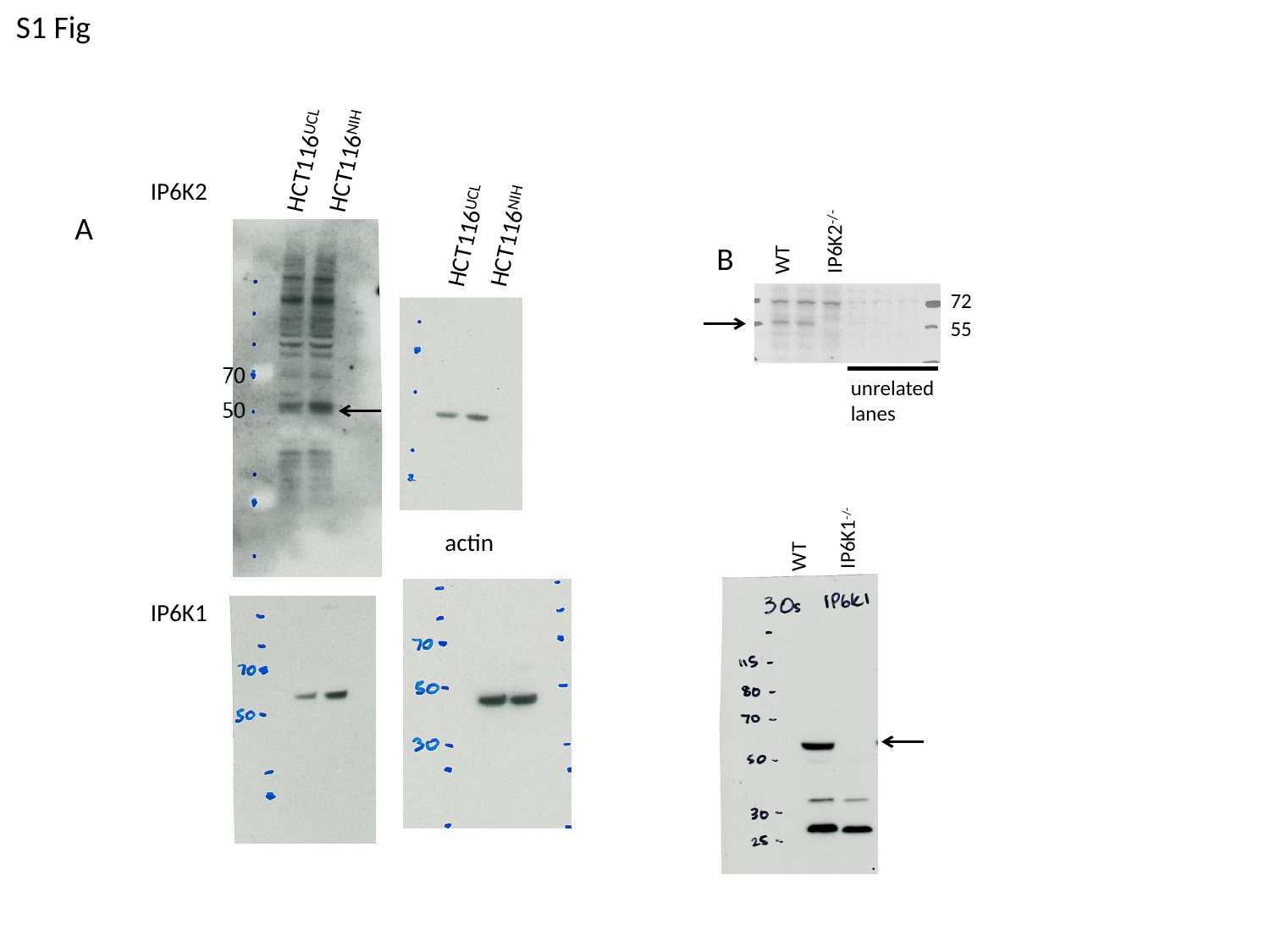

S1 Fig
HCT116NIH
HCT116UCL
IP6K2
actin
IP6K1
A
HCT116NIH
HCT116UCL
IP6K2-/-
B
WT
72
55
unrelated
lanes
IP6K1-/-
WT

Supplement: S1 Fig — Panel A, complete blots are shown for the Western analyses of levels of IP6K1, IP6K2 and actin as depicted in Fig 3A of the main text. Panel B, validation of the band detected by the anti-IP6K2 antibody (using an extract prepared from IP6K2-/- HCT116 cells) and the anti-IP6K1 antibody (using an extract prepared from IP6K1-/- MEF cells). (PPTX) [file pone.0165286.s001.pptx]

## Slide 1
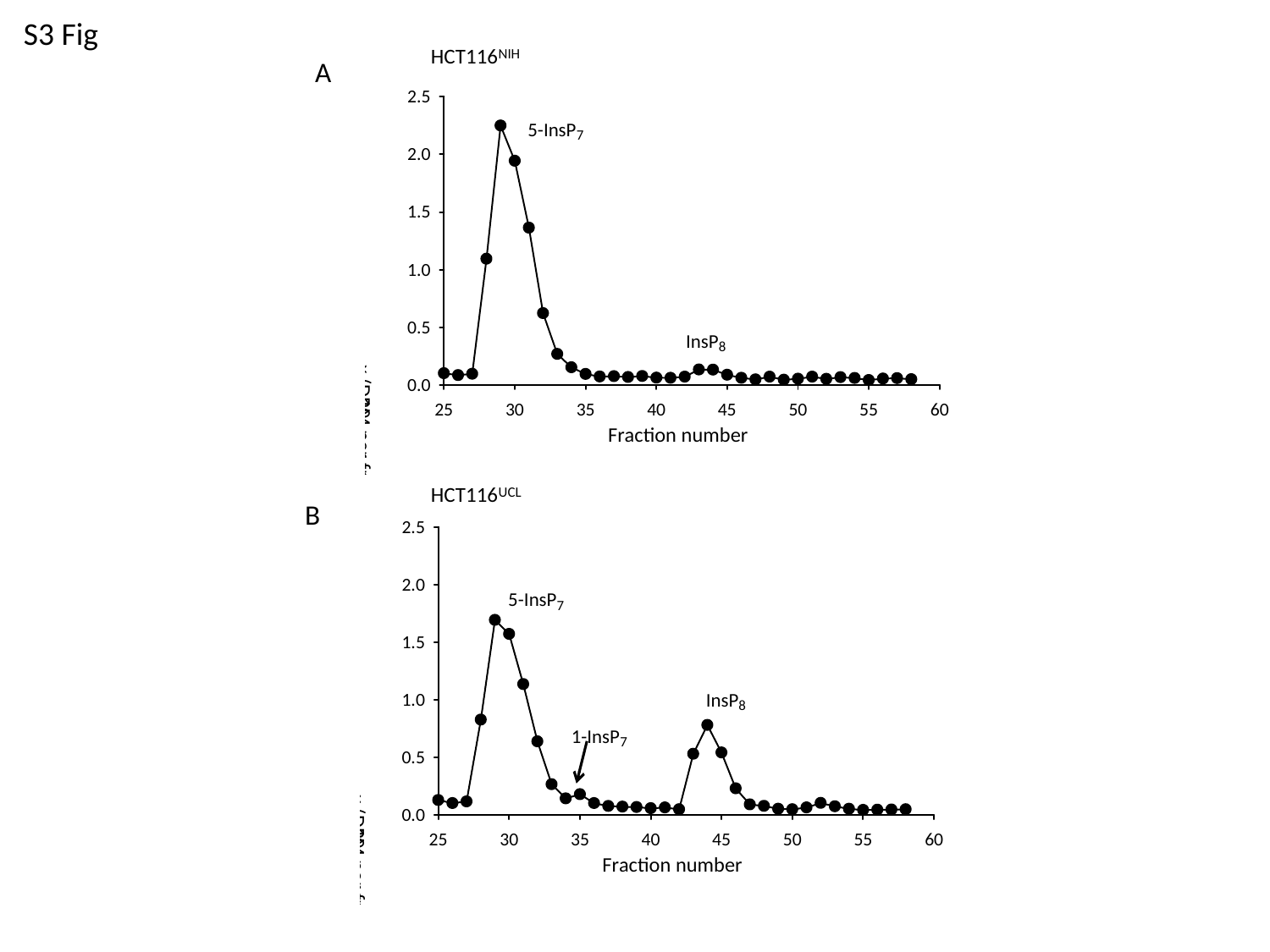

S3 Fig
HCT116NIH
A
5-InsP7
InsP8
HCT116UCL
B
5-InsP7
InsP8
1-InsP7

Supplement: S3 Fig — Extracts of [3H]inositol-labeled HCT116NIH cells (Panel A) and HCT116UCL cells (Panel B) were prepared in parallel and analyzed by CarboPac HPLC. The DPM in each fraction were normalized to the DPM of the [3H]inositol lipids. Only InsP7 and InsP8 peaks are shown. This experiment was performed six times. In the experiment shown, 1-InsP7 is only discernable in the HCT116UCL cells. Fig 5 in the main text shows a separate experimental pair in which 1-InsP7 was only observed in the HCT116NIH cells. (PPTX) [file pone.0165286.s003.pptx]

## Slide 1
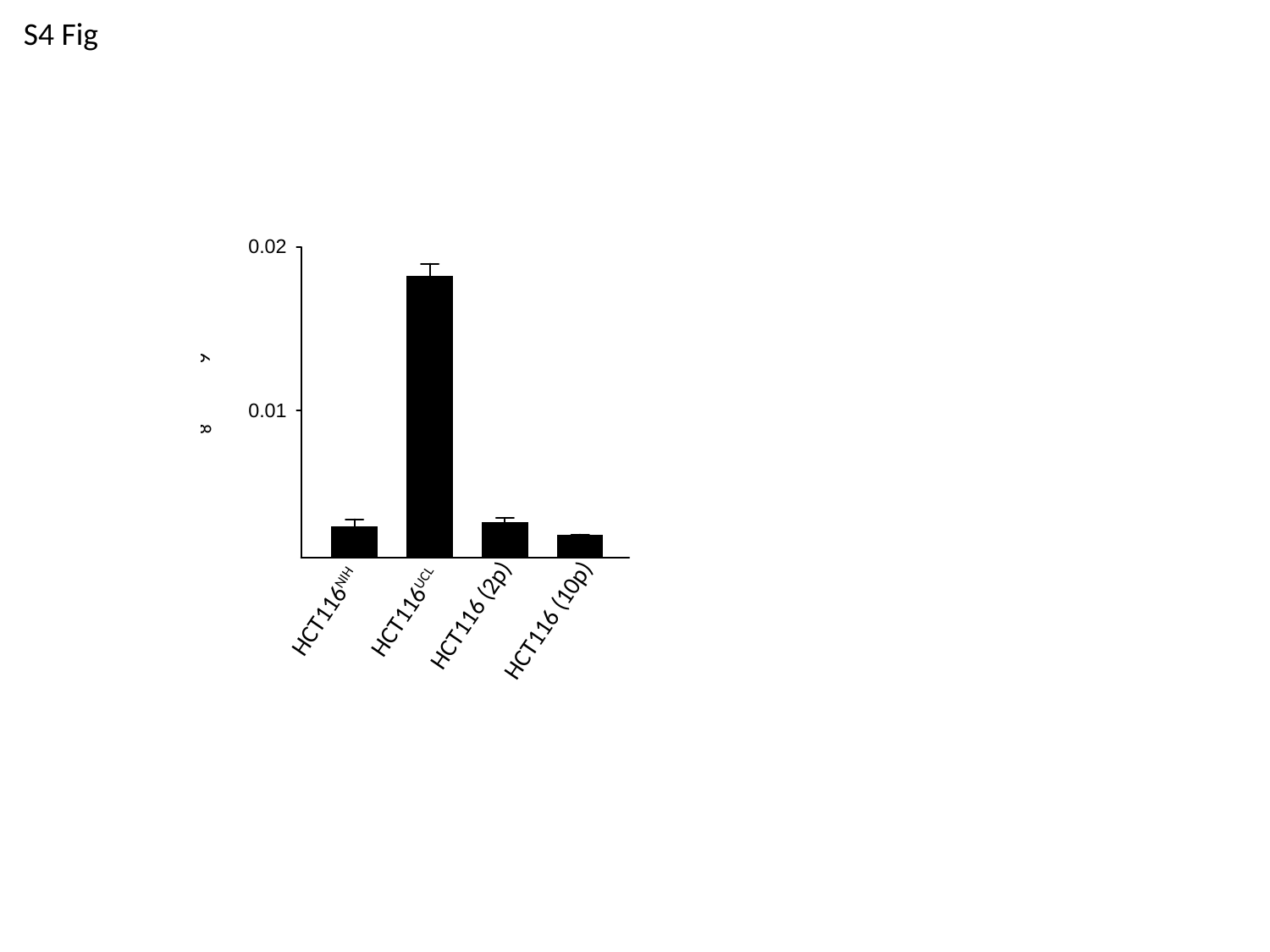

S4 Fig
HCT116NIH
HCT116UCL
HCT116 (2p)
HCT116 (10p)

Supplement: S4 Fig — CarboPac HPLC was used to quantify [3H]InsP8 levels in extracts prepared from [3H]inositol-labeled HCT116NIH cells, HCT116UCL cells, and also parental HCT116 cells that were procured directly from ATCC and analyzed after 2 passages (“2p”) and 10 passages (“10p”). [3H]InsP8 levels are normalized to those of [3H]InsP6. (PPTX) [file pone.0165286.s004.pptx]
