# Supplementary material for: Inositol Pyrophosphate Profiling of Two HCT116 Cell Lines Uncovers Variation in InsP8 Levels
Source: PLoS One. 2016 Oct 27;11(10):e0165286. doi: 10.1371/journal.pone.0165286 (PMC5082907; doi:10.1371/journal.pone.0165286)
Supplement: S2 Fig — Panels A, B, complete blots are shown for the Western analyses of levels of PPIP5K2, PPIP5K1 and actin as depicted in Fig 3A of the main text. Panel C, validation of the PPIP5K1 and PPIP5K2 band detected by the anti-PPIP5K2 antibody, in a single blot with two different exposure times. K1KO and K2KO lanes show extracts prepared from cells in which either PPIP5K1 or PPIP5K2 expression, respectively, was eliminated using CRISPR. Single-guide RNAs(sgRNA) with sequences 5’-CCCCTTTCTTATCAATGATCTGG-3’ and 5’-CGGTTCAAAATAGCATAACGAGG-3’ were designed to target PPIP5K1 exon 4 and PPIP5K2 exon 5 respectively. Vector expressing both cas9 and sgRNA was obtained from Addgene (PX458). PPIP5Ks KO cells were generated following the protocol as described: Genome engineering using the CRISPR-Cas9 system. Nat Protoc. 2013 Nov; 8(11): 2281-308. doi: 10.1038/nprot.2013.143. Epub 2013 Oct 24. (PPTX) [file pone.0165286.s002.pptx]

## Slide 1
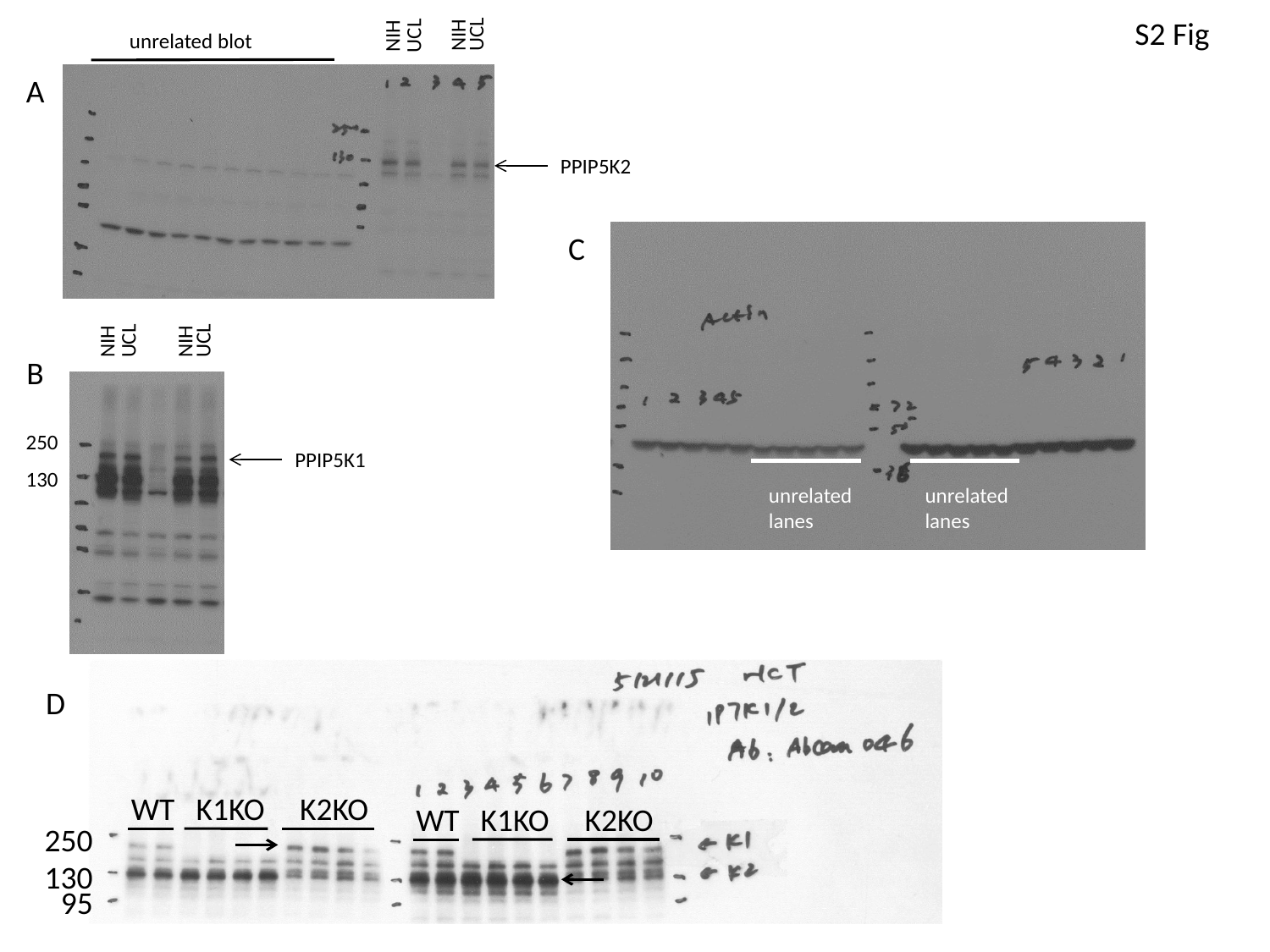

S2 Fig
UCL
UCL
NIH
NIH
unrelated blot
A
PPIP5K2
C
UCL
UCL
NIH
NIH
250
PPIP5K1
130
B
unrelated
lanes
unrelated
lanes
D
WT
K1KO
K2KO
WT
K2KO
K1KO
250
130
95
